# Accumulation, Depuration, and Biological Effects of Polystyrene Microplastic Spheres and Adsorbed Cadmium and Benzo(a)pyrene on the Mussel *Mytilus galloprovincialis*

**DOI:** 10.3390/toxics10010018

**Published:** 2022-01-05

**Authors:** Rebecca von Hellfeld, María Zarzuelo, Beñat Zaldibar, Miren P. Cajaraville, Amaia Orbea

**Affiliations:** 1 CBET Research Group, Department of Zoology and Animal Cell Biology, Biotechnology PiE and Science and Technology Faculty, Research Centre for Experimental Marine Biology, University of the Basque Country (UPV/EHU), Sarriena z/g, 48940 Leioa, Basque Country, Spain; rebecca.vonhellfeld@abdn.ac.uk (R.v.H.); mariazarzuelo1997@gmail.com (M.Z.); benat.zaldibar@ehu.eus (B.Z.); mirenp.cajaraville@ehu.eus (M.P.C.); 2 School of Biological Sciences, University of Aberdeen, 23 St. Machar Drive, Aberdeen AB24 3UU, UK

**Keywords:** polystyrene microplastics, size-dependent uptake, vectors, cadmium, benzo(a)pyrene, mussels

## Abstract

Filter feeders are target species for microplastic (MP) pollution, as particles can accumulate in the digestive system, disturbing feeding processes and becoming internalized in tissues. MPs may also carry pathogens or pollutants present in the environment. This work assessed the influence of polystyrene (PS) MP size and concentration on accumulation and depuration time and the role of MPs as vectors for metallic (Cd) and organic (benzo(a)pyrene, BaP) pollutants. One-day exposure to pristine MPs induced a concentration-dependent accumulation in the digestive gland (in the stomach and duct lumen), and after 3-day depuration, 45 µm MPs appeared between gill filaments, while 4.5 µm MPs also occurred within gill filaments. After 3-day exposure to contaminated 4.5 µm MPs, mussels showed increased BaP levels whilst Cd accumulation did not occur. Here, PS showed higher affinity to BaP than to Cd. Three-day exposure to pristine or contaminated MPs did not provoke significant alterations in antioxidant and peroxisomal enzyme activities in the gills and digestive gland nor in lysosomal membrane stability. Exposure to dissolved contaminants and to MP-BaP caused histological alterations in the digestive gland. In conclusion, these short-term studies suggest that MPs are ingested and internalized in a size-dependent manner and act as carriers of the persistent organic pollutant BaP.

## 1. Introduction

In 2019, world plastic production reached 368 million tons [1], and the lack of efficient plastic management has led to severe consequences for ecosystems [2]. Moreover, the plethora of paths through which plastic enters the marine environment has allowed large quantities of plastic to accumulate [3]. The different types and sizes of plastic [2], such as water bottles, bags and industrially produced plastic pellets and microparticles [4], have been found to affect all trophic levels [5,6]. Microplastics (MPs) are defined as plastic particles with a diameter of less than 5 mm [7] and, according to the European Marine Strategy Framework Directive (MSFD) Technical Subgroup on Marine Litter, should be further classified as small MPs (<1 mm) and large MPs (1–5 mm) [8]. These minute particles easily disperse in the water column and are frequently found in sediment samples [9] and in biota [10].

Interactions with the environment alter the particles’ structure, resulting in changing surface properties [11]. Over time, these processes increase their porosity, charge and roughness, leading to an increase in accumulation of other compounds present in the environment [12,13,14]. It has been found that the smaller a particle is, the larger its surface area-to-volume ratio will be, leading to greater contaminant adsorption [15]. It has further been reported that over 70% of chemicals listed as priority pollutants by the United States EPA bind with plastic debris [14]. Thus, the high capacity of plastic debris for adsorbing pollutants poses an additional threat to marine wildlife [16], as the adsorbed contaminants may desorb once the particle has been ingested [17]. The bioaccumulation potential of some of these potentially adsorbed contaminants can thus be seen throughout ecosystems [18], which may lead to the transfer of pollutants across generations [19,20] or alterations at subcellular level [21,22]. This underlines the importance of understanding the vector potential of MPs in order to accurately predict the risk of water borne contaminants in conjunction with the increasing pollution of marine waters with plastic particles [1].

Mussels are filter-feeding sessile organisms tolerant to salinity changes and other stressors. Moreover, due to their high water filtration rate and low metabolic activity, they accumulate dissolved and particulate pollutants at levels higher than those present in the water column [22]. This makes them an excellent species for MP research, allowing for comparability and transferability of results [23,24,25,26]. One widely distributed species is the Mediterranean mussel *Mytilus galloprovincialis*, found along almost every coastline worldwide [27], inhabiting the zone between the rocky shore and sandy bottom [28], and are thus widely used as sentinel organisms in pollution monitoring [24,29]. MPs were found to enter bivalves through the gill filaments, thus being the initial entry point for particulate pollutants and associated contaminants [4,23,30,31] and making it an organ of interest in biomarker studies. The ingested particles then move towards the mouth and enter the digestive gland [23,30,32] or even reach the gonad tissue [33]. Despite the relevant amount of data regarding MP particles entering bivalves, more information is needed on the retention time and depuration capacity. This is especially important, since the longer the retention time of these particles, the more likely it is that they will be transferred to the next trophic level upon consumption [34], as well as the more time chemicals and other compounds have to potentially desorb from the particles [23,32,35]. A multitude of studies have been conducted on the effects of pristine and contaminated plastic particles on the health of marine mussels [36,37], as well as the thus resulting health effect for humans [38] for different compounds.

Rios Mendoza et al. [39] assessed the concentration of pollutants sorbed to plastic debris in the North Pacific gyre and found that almost 80% of the debris they collected contained polycyclic aromatic hydrocarbons (PAHs), with concentrations ranging from a few to thousands of parts per billion (ppb). Benzo(a)pyrene (BaP), a PAH that originates from tar, burning wood, exhaust fumes and fumes from brunt organic material has been widely used as a model compound in aquatic toxicology [40] and, more recently, to assess the potential of MPs as carriers of hydrophobic pollutants. BaP reacts and binds to DNA, making it a highly efficient mutagen and carcinogen [41]. It is the only PAH classified as a recognized carcinogen by the International Agency for Research on Cancer (IARC) [42] and has routinely been employed as model contaminant for this group. It has also been classified as a candidate for being a substance “of very high concern” in the Registration, Evaluation, Authorization, and Restriction of Chemicals (REACH). Exposure has been found to induce CYP1A and morphological changes in gill tissue [42], as well as being found to accumulate through the food chain [43]. Direct exposure of mussels *M. galloprovincialis* to 20–25 µm low density PE (LDPE) MPs led to particle localization in the haemolymph and gills, as well as digestive tissue, whilst BaP contaminated MPs led to significant alterations of the immune system [44]. Dietary exposure to BaP contaminated polystyrene (PS) MPs also caused an exposure-time dependent increase in BaP concentration in mussels, particularly when sorbed to smaller MPs (0.5 µm versus 4.5 µm) [33]. Overall, BaP-contaminated MPs were more toxic than pristine MPs, according to haemocyte viability, catalase activity, and to the quantitative structure of digestive tubule epithelium.

Cadmium (Cd) is known to be persistent in the environment and to bioaccumulate up the food chain, similar to many lipophilic metals [45]. This makes it a suitable model contaminant to examine the vector potential of MPs for metals. Although metals readily adsorb to MPs, the co-exposure to copper or silver contaminated PE MPs was found to have no additional effect on marine microalgae [46] and zebrafish [47], whilst exposure to the metals alone had negative impacts on the individuals. However, synergistic sublethal toxicity of Cd and PS MPs at high levels (1, 5, 10 mg/L) was reported in zebrafish embryos [48]. In a study carried out in Vancouver (Canada), up to 7% of beached MPs were found to have adsorbed Cd [49].

Biomarkers, such as the activity of the antioxidant enzymes catalase (CAT) and superoxide dismutase (SOD), are often measured as indicators of the potential oxidative stress caused by pollutants, whilst the peroxisomal enzyme acyl-CoA oxidase (AOX) is assessed as a biomarker of exposure to organic contaminants [50]. The biomarker approach has also been applied to detecting deleterious effects caused by exposure to pristine and contaminated plastics on the health of aquatic organisms, such as mussels and copepods [33,44,51]. An established metal exposure biomarker is the quantification of lysosomal accumulation of metals in mussel tissues through autometallography [52]. Effects on cellular and tissue level can be determined through the assessment of the lysosomal membrane stability [32,44] and the histological structure of the digestive gland [33], respectively.

The present work aims (1) to examine the accumulation, depuration time and tissue distribution of 45 and 4.5 µm polystyrene MPs at different concentrations in the mussel *M. galloprovincialis* through histological analysis after short term exposure, and (2) to determine the fate and impact of adsorbed BaP and Cd on mussels through analytical chemistry and a battery of biomarkers.

## 2. Materials and Methods

### 2.1. Mussels

Mussels with a shell length between 3.5 and 4.5 cm were collected in the estuary of Plentzia, Basque Country (Bay of Biscay, 43°24′ N; 2°56′ W), considered as a reference site [50], during low tide in March (for experiment 1) and April 2016 (for experiment 2). Individuals were rinsed with water from the sampling location and transferred to the laboratory within the hour. Mussels for the experimental exposures to MPs were kept in an aerated tank with continuous filtered natural seawater supply for five days to acclimatize. Seawater from Plentzia was naturally filtered by sand in the uptake wells aided with a pump that sent the water to the Marine Station. Seawater gas balance was controlled in the station and then passed through a decantation/inertial tank and filtered (particle size ≤ 3 µm). Mussels were fed twice daily with Sera Marin “Coraliquid” (Sera, Heinsberg, Germany), and routine health checks were performed every morning, with no mortality observed during the acclimatization period.

### 2.2. Microplastics

PS spheres of 45 and 4.5 µm in diameter in a commercial solution (2.5% solids in deionised water with residual surfactant) were purchased from Polyscience Inc. (Badener, Germany). According to manufacturer’s information, particles showed slight anionic charge and were monodispersed with a maximum coefficient of variation of 10% and 7% for the 45 and 4.5 µm particles, respectively.

### 2.3. Experiment 1: 1-Day Exposure and 3-Day Depuration of Pristine MPs

After the acclimatization period, mussels sampled in March were randomly distributed into 14 high density polyethylene containers (Deltalab, Barcelona, Spain) containing one litre of filtered natural seawater and exposed for 1 day to 1 (C1), 100 (C2) and 1000 (C3) particles/mL of PS microspheres of 45 and 4.5 µm in diameter, equivalent to 0.05, 5 and 50 mg/L for 45 µm MPs and 0.05, 5 and 50 µg/L for 4.5 µm MPs. In addition, a control group was maintained unexposed, and all treatments were run in duplicate. The selected MP particle concentrations of the present publication are within reported environmentally relevant concentrations (e.g., 1770 particles/L found in the southern North Sea [53]).

During the experiment, mussels were fed twice with “Coraliquid”. After 1 day of exposure (E), 5 organisms per replicate of each treatment group were collected, cleaned, and processed for the histological localization of MPs. The remaining exposed mussels were then transferred back into uncontaminated water to allow depuration. After 1 (D1), 2 (D2) and 3 days (D3) of depuration 5 organisms per replicate of each exposure group were sampled. From each individual, a portion of the digestive gland and of the gill tissue was placed in histology cassettes and fixed in 10% buffered formalin for paraffin embedding. Tissue dehydration and infiltration steps were performed using n-butyl alcohol [33]. Paraffin embedded tissues were cut using a RM2125RT microtome (Leica Microsystems GmbH, Wetzlar, Germany) into 5 µm thickness sections. Three histological sections with a distance between them of at least 15 µm were collected onto microscopy slides from each individual and tissue. Sections were dewaxed utilizing n-butyl alcohol and stained with hematoxylin and eosin (H&E). Slides were mounted with Kaiser’s glycerin gelatin (Merck KGaA, Darmstadt, Germany). Sections were examined for MP localization and photographed using an Olympus BX50 microscope (Olympus, Tokyo, Japan).

### 2.4. Experiment 2: 3-Day Exposure to Pristine and Contaminant Adsorbed MPs

Particles of 4.5 µm were contaminated with BaP or Cd after Batel et al. [43]. The procedure was repeated daily prior to dosing. The MPs were incubated in the dark in 10 mL of a 1 µM BaP or Cd water solution (252.3 and 112.4 µg/L, respectively). BaP was initially dissolved in DMSO and then diluted in MilliQ water to reach a final DMSO concentration in the incubation medium of 0.01%, a concentration that was found to induce no alterations in biomarker responses in mussels [54]. Cd 1 µM was prepared from CdCl_2_. After 1 day in the orbital shaker (Rotabit, Selecta, Barcelona, Spain), the MP suspension was filtered through a 0.45 µm sterile filter (Merck Millipore, Darmstadt, Germany). MPs retained in the filter were washed twice with dH_2_O and recovered with 10 mL dH_2_O. The MPs were then resuspended in 40 mL dH_2_O and added to the aquaria.

After the initial acclimatization period, mussels sampled in April were distributed in glass aquaria of 10 L. For 3 days, organisms were either exposed to 1000 particles/mL pristine 4.5 µm MPs, 1000 particles/mL plastic particles previously exposed to 1 µM Cd (MP-Cd) or BaP (MP-BaP), 1 µM dissolved Cd or BaP without plastic particles, or filtered natural seawater as control group. Particle concentration was based on the outcome of experiment 1. For the dissolved pollutant exposure, the glass tanks were pre-exposed for 24 h to allow for saturation. The water in the aquaria was fully renewed fully every 24 h prior to redosing. Mussels were fed and monitored as described above.

Every day, 30 min and 24 h after dosing, water samples were collected from the aquaria to monitor Cd and BaP concentrations. After 3 days, mussel samples were cleaned and collected for (1) chemical analyses of Cd and BaP concentrations for which whole mussels were frozen and stored at −20 °C until analysis; (2) activity of antioxidant and peroxisomal enzymes catalase (CAT), superoxide dismutase (SOD) and acyl-CoA oxidase (AOX), for which the digestive gland and gills of mussels were dissected, frozen in liquid nitrogen and stored at −80 °C until analysis; (3) the evaluation of the lysosomal membrane stability, for which half of the digestive gland was frozen in liquid nitrogen and stored at −80 °C until cryo-sectioning¸ and (4) MP localization, quantitative assessment of the structure of the digestive gland, and metal localization and distribution after autometallographical staining, for which the other half of the digestive gland was placed in histology cassettes and fixed in 10% buffered formalin for paraffin embedding, as described above.

#### 2.4.1. Chemical Analysis of the Mussel and Water Samples

Chemical analyses of water and mussel samples were carried out in the General Research Services (SGIker) at the University of the Basque Country. Sixty mussels sampled in March were used for chemical analysis of PAHs and metals to ascertain the background concentration of contaminants.

For the analysis of metal body burdens, mussel samples were dried in pools (5 replicates) at 120 °C for 48 h, weighted and digested in HNO_3_ Tracepur^®^ 69% (Panreac, Barcelona, Spain). Once the concentrated acid was evaporated, pellets were resuspended in 0.01 M HNO_3_ Tracepur^®^ and quantified. The metal analysis was carried out by inductively coupled plasma atomic emission spectrometry (ICP-AES, Horiba Yobin Yvon Activa, Horiba Japan Domestic Group, Kyoto, Japan) for Fe and Zn and by ICP-mass spectrometry (ICP-MS; Agilent 7700, Agilent Technologies, Santa Clara, CA, USA) for Cr, Ni, Cu, Cd and Pb. The certified reference material NIST 2976 was used for quality control. A detection limit of 13 ng/g for Fe, Cr, Ni, Cu and Cd; 0.1 µg/g for Pb, and 2.0 µg/g for Zn was determined.

The analysis of the 16 EPA PAHs was performed by gas chromatography and mass spectrometry in 5 replicates. Approximately 1 g freeze-dried samples were extracted with acetone in a microwave oven (MARX, CEM, Matthews, NC, USA) and cleaned up by solid phase extraction (SPE) using Millipore cartridges (Merck Millipore). Six deuterated PAHs were added to the samples to monitor the recovery efficiency and two blank samples were run in parallel. The extracts were analysed in a 6890 Agilent gas chromatograph coupled to a 5975C Agilent mass spectrometer (Agilent Technologies, Avondale, PA, USA). A detection limit of 1 ng/g was determined for all PAHs, except for acenaphthalene (0.1 ng/g) and naphthalene (10 ng/g).

During the second experiment, water samples were collected for chemical analyses. For the measurement of Cd concentration, 50 mL of water from the MP, MP-Cd and Cd groups (3 replicates of each) were collected. All water samples were filtered through a PES membrane (0.2 µm), acidified with ultra-pure hydrochloric acid (1% *v*/*v*) and stored at 4 °C for no longer than two days before analysis. Analyses were carried out in an ICP-MS Agilent 7700 spectrophotometer as mentioned above. For the analysis of BaP, 500 mL of water were collected (3 replicates of each) in glass bottles from the MP, MP-BaP and BaP groups and stored at 4 °C in the dark until being analysed. These water samples were mixed with propanol and, after adding deuterated BaP as internal standard, samples were extracted by SPE and analysed using the same equipment described above. At the end of the exposure, 10 mussels were also collected and frozen whole, to assess Cd and BaP concentrations in the same groups mentioned for the water samples. Five pooled samples were used for the assessment of Cd concentration and two pooled samples for BaP concentration. Analyses of mussel samples were performed as described above.

#### 2.4.2. Biochemical Analysis of the Antioxidant and Peroxisomal Enzyme Activity

Digestive glands or gills of six individuals per experimental group were homogenized in 3 mL of TVBE buffer (1 mM sodium bicarbonate, 1 mM EDTA, 0.1% ethanol and 0.01% Triton X-100, pH 7.6) per gram of tissue using a glass-Teflon^®^ homogenizer (Potter S, B. Braun Melsungen AG, Melsungen, Germany) in an ice water-cooled bath. Homogenized samples were centrifuged at 500× *g* for 15 min in a Beckman Coulter Allegra 25R Centrifuge (Beckman Coulter Life Sciences, Indianapolis, IN, USA). The pellet was discarded, and 50 µL aliquots of the supernatant were frozen and stored for the measurement of AOX activity and protein concentration. The remaining supernatant was centrifuged at 12,000× *g* for 45 min. The pellet (mitochondrial fraction) was resuspended in 1 mL homogenization buffer per gram of initial tissue and frozen for later determination of CAT activity and protein concentration. The supernatant (S12 fraction) was divided in three aliquots and frozen for the measurement of CAT and SOD activity, and protein concentration.

Peroxisomal AOX activity was measured as described by Small et al. [55]. The assay is based on the H_2_O_2_-dependent oxidation of dichlorofluorescein catalysed by an exogenous peroxidase using 30 mM palmitoyl-CoA as substrate. CAT activity was calculated as the sum of the activities assessed in the mitochondrial and S12 fractions by measuring the disappearance of H_2_O_2_ at 240 nm (extinction coefficient 40 M^−1^ cm^−1^) in a Shimadzu UV-1800 spectrophotometer (Shimadzu, Columbia, SC, USA) using 50 mM H_2_O_2_ as substrate in 80 mM potassium phosphate buffer (pH 7) [56]. SOD activity was determined in the S12 fraction at 550 nm by measuring the inhibition of cytochrome c reduction by superoxide generated by the xanthine oxidase/hypoxanthine system in an assay mixture that contained 50 mM potassium phosphate buffer plus 0.1 mM EDTA (pH 7.8), 50 mM hypoxanthine, 1.87 mU mL^−1^ xanthine oxidase and 10 mM cytochrome c [57]. One SOD unit was defined as the amount of enzyme that inhibits the rate of cytochrome c reduction by 50%. Protein concentration was measured in all fractions using the Quick Start™ Bradford Protein Assay Kit 3 (Bio Rad Life Sciences, Hercules, CA, USA).

#### 2.4.3. Lysosomal Membrane Stability

The lysosomal membrane stability (LMS) test was performed according to a standardized protocol [58]. Serial tissue sections (10 μm thick) of 10 individuals per experimental group were cut in a Leica CM 3050S cryostat (Leica) and stored at −40 °C until required for staining. Briefly, the lysosomal membrane was destabilized at 37 °C for different periods of time (0, 3, 5, 10, 15, 20, 30 and 40 min) using 0.1 M sodium citrate buffer (pH 4.5) plus 2.5% NaCl. Then, sections were incubated for 20 min at 37 °C in 0.1 M citrate buffer (pH 4.5) containing 2.5% NaCl, 0.04% naphthol AS-BI N-acetyl-β-D-glucosaminide dissolved in 2-methoxiethanol (Merck KGaA, Darmstadt, Germany) and 7% Polypep^®^ (Merck KGaA) as a section stabilizer. After incubation, sections were rinsed in a saline solution (3% NaCl) at 37 °C for 2 min and introduced into 0.1 M phosphate buffer (pH 7.4) containing 0.1% diazonium dye Fast Violet B salt, at room temperature for 10 min. Slides were rinsed in running tap water for 5 min, fixed for 10 min in 10% formaldehyde containing 2% calcium acetate at 4 °C and rinsed in distilled water. Finally, slides were mounted in Kaiser’s glycerol gelatine. The determination of lysosomal membrane stability was based on the time of acid labialization required to produce maximum lysosomal staining. The labialization period (LP) was assessed under an Olympus BX-50 light microscope using an objective lens of 40× magnification. Each digestive gland was divided into four sections for the analysis to obtain the mean value of LP.

#### 2.4.4. Tissue Metal Accumulation after Autometallography

A set of paraffin sections (10 individuals per experimental group) was stained with the BBI Solutions Silver enhancer kit (TAAB Laboratories Equipment, Aldermaston, UK) to assess the presence of metals in histological sections of the gills and digestive gland shown as black silver deposits (BSDs). Five fields of each section were photographed using the 40× magnification objective and the percentage of the digestive tissue area occupied by BSDs was measured by image analysis with the aid of ImageJ software (version1.50i, National Institutes of Health, USA).

#### 2.4.5. Quantitative Histological Analysis

Changes in digestive gland structure of 10 individuals per experimental group were assessed by means of quantitative histology in paraffin sections stained with H&E. Volume density of basophilic cells (*VvBAS*), mean epithelium thickness (*MET*), mean luminal radius (*MLR*) and mean diverticular radius (*MDR*) of digestive gland tubules were determined applying a stereological procedure [59,60]. A M-168 Weibel multipurpose test system was superimposed to microscopic images (20× objective) with the aid of a drawing tube attached to an Olympus BX51 microscope and hits on basophilic cells (b) digestive cells (d), diverticular lumen (l) and interstitial connective tissue (c) were recorded. The following equations were applied:

(1) $$VvBAS=\frac{b}{\left(d+b\right)}$$

(2) $$MET=\frac{2d\surd \pi }{\left(\surd \left(\left(b+d\right)+\sqrt{1}\right)\right)}$$

(3) $$MLR=\surd \frac{1}{\pi }$$

(4) $$MDR=\sqrt{\frac{\left(b+d+1\right)}{\pi }}$$

*MLR*/*MET* and *MET*/*MDR* ratios were calculated as well, along with connective to diverticula (CTD) ratio, which was calculated as CTD = *c*/(*b* + *d* + l) [59].

### 2.5. Statistics

The normal distribution and homogeneity of variances of each dataset was assessed with the Shapiro test and the Levene’s test, respectively. For data following a normal distribution and with homogeneous variances, one-way ANOVA was applied followed by the Tukey’s HSD post hoc test. The non-normal/non-homogenous data were assessed using one-way Kruskal–Wallis test followed by Dunn’s test. Analyses were performed using SPSS Standard (version 21.0.0 for Mac OS X) and statistical significance was established at *p* < 0.05.

## 3. Results

### 3.1. Accumulation and Depuration of MPs in Mussel Tissue

None of the unexposed control organisms showed any plastic particle in the histological assessment. In experiment 1, after 1 day of exposure, the abundance of the 45 µm sized particles in the digestive gland increased in a concentration dependent manner, with particles present in the digestive gland of the 50% of the individuals exposed to the lowest concentration (1 particle/mL) and in the 100% of organisms exposed to 100 and 1000 particles/mL (Table 1). Moreover, increasing concentrations also led to longer retention times within the mussels. Amounts of 20% and 40% of the mussels exposed to the two highest concentrations retained MPs in the digestive gland by the third depuration day (Table 1). At the lowest concentration, no particles were found in any structure of the digestive gland after ≤3 days of depuration.

**Figure 1:**
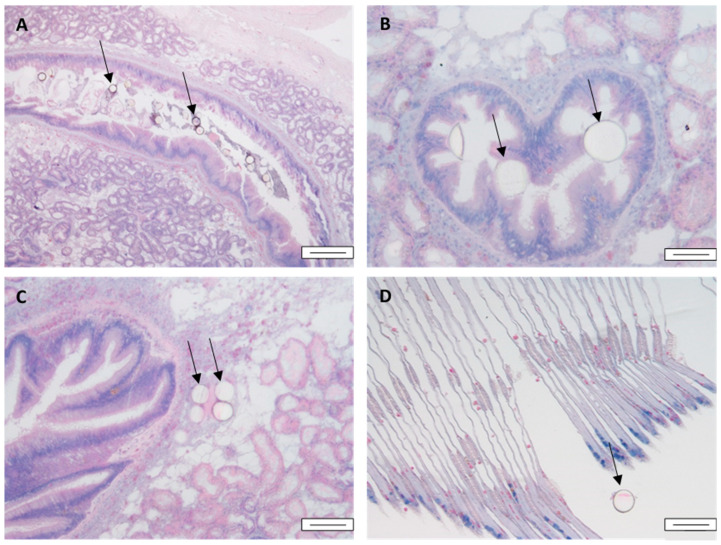
Micrographs of H&E-stained sections of digestive gland and gills of mussels after 1 day of exposure to pristine 45 µm MPs. (**A**) MPs in the lumen of the stomach after exposure to 100 particles/mL; (**B**) MPs in the lumen of a duct after exposure to 1000 particles/mL; (**C**) MPs in the connective tissue after exposure to 1000 particles/mL; (**D**) MPs outside a gill filament after exposure to 100 particles/mL. Black arrows point to MP particles. Scale bars: (**A**) 200 µm, (**B**) 50 µm, (**C**,**D**) 100 µm.

In mussels exposed to 100 particles/mL, most particles appeared in the lumen of the stomach lumen (Figure 1A), duct, and tubule, and the connective tissue. Exposure to 1000 particles/mL led to a higher abundance in all sample regions (Figure 1B,C, Table 1). By the third day of depuration, MPs remained mostly in the stomach lumen, with few observed in digestive duct lumen (Table 1). MPs of 45 µm were observed in the gills less frequently than in the digestive gland, with 40% being the highest observed prevalence prior to depuration of the highest exposure concentration group (1000 particles/mL), and particles were rarely observed within the gills after 1 day of depuration. However, they were found both within and outside the gill filaments (Figure 1D). Exposure to 1 particle/mL led to some particles observed after 1 day of exposure outside of the filaments, whilst no particles were observed within the structure.

Particles of 4.5 µm were found in both the digestive gland and the gills in almost all treated groups, even after the full depuration period (Table 1). The highest prevalence of 4.5 µm MPs in mussels was observed after 1-day exposure. After 2 days of depuration, 40 to 60% of organisms exposed to 100 and 1000 particles/mL still showed particles in the gill and digestive gland samples. Overall, a concentration-dependent increase in abundance and dispersal was found in the digestive gland The digestive gland samples exhibited a steeper decrease in affected individuals with depuration time than the gill samples, with a reduction of 50 to 60% when exposed to 1–1000 particles/mL. In the digestive gland, the 4.5 µm particles were found exclusively in the stomach and duct lumen at the lowest exposure concentration and the organisms had depurated completely by the final day. When exposed to 1000 particles/mL, MPs were found in stomach, duct, and tubule lumen by the end of the experiment.

Gill depuration for organisms exposed to 4.5 µm particles was between 30 and 50% with increasing concentration. Here, the particles were mainly located between the filaments or in the frontal area of the gill filaments. Throughout all exposure concentrations, some particles were observed within the gill filaments, however, with slight decreases noted over the depuration time.

**Figure 2:**
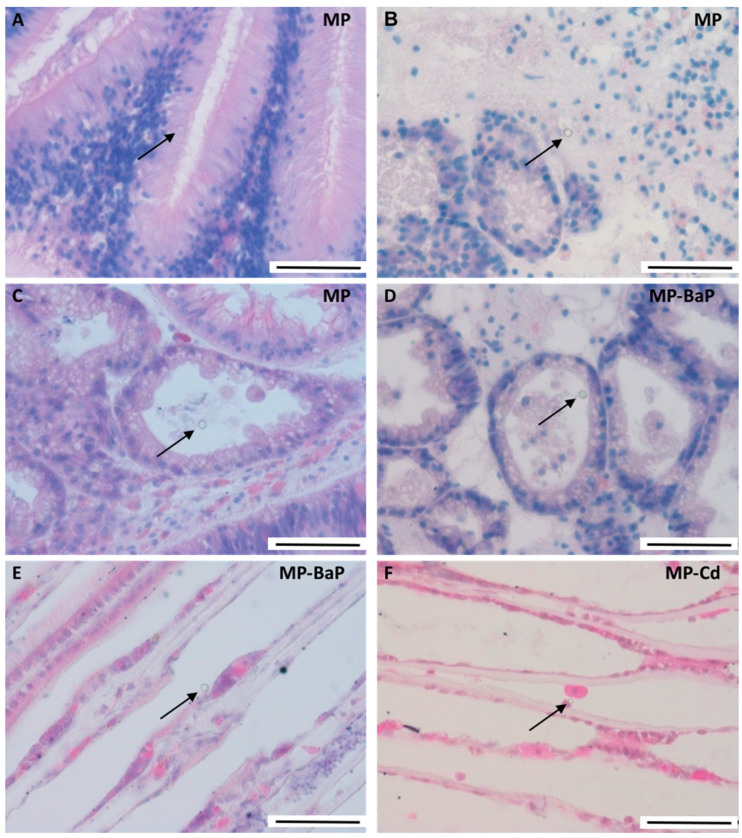
Micrographs of H&E-stained sections of digestive gland and gills of mussels after 3-day exposure to 1000 particles/mL pristine and contaminated 4.5 µm MPs. (**A**) MP in the stomach epithelium after exposure to pristine particles; (**B**) MP in the connective tissue surrounding the digestive tubules after exposure to pristine particles; (**C**) MP in the lumen of a digestive tubule after exposure to pristine particles; (**D**) MP in the lumen of a digestive tubule after exposure to MP-BaP; (**E**) MP over a gill filament after exposure to MP-BaP; (**F**) MP inside a gill filament after exposure to MP-Cd. Black arrows point to MP particles. Scale bars: 50 µm.

In experiment 2, mussels exposed for 3 days to pristine 4.5 µm MPs and to MPs contaminated with Cd and BaP showed the same tissue distribution of MPs described above (Figure 2) but, in this case, some particles were also observed within the stomach epithelium (Figure 2A).

### 3.2. Metal and PAH Accumulation in Mussels and Concentration in Exposure Media

The background contamination by metals and PAHs of the mussels sampled in Plentzia can be found in Table 2. PAHs such as acenaphthalene, indenopyrene and dibenzo(a,h)anthracene were found at levels below the detection limit (bdl) in some of the analysed samples. The Cd concentration detected in field mussels was similar to the Cd concentration measured in mussels exposed to pristine MPs and in mussels exposed to MP-Cd (Table 2 and Table 3). However, organisms exposed to dissolved Cd for 3 days showed a Cd concentration 50 times greater than that of mussels exposed to pristine plastics. Field mussels showed slightly higher concentration of BaP than mussels exposed to pristine MPs, possibly due to the acclimatization period the exposed organisms were given after sampling, which the organisms from the field did not have. Whilst plastic-bound Cd did not increase the tissue Cd concentration, plastic-bound BaP notably increased the BaP concentration in the tissue samples, indicating that the plastic particles acted as vehicles for BaP to mussels. The highest concentration of BaP was observed in mussels exposed to BaP dissolved in water (Table 3).

The analysis of the water samples collected from the exposure tanks (Table 4) showed that the Cd concentration was below the detection limit in the aquaria containing pristine MPs and MP-Cd. Water samples from the tanks of the Cd-exposed organisms indicated that the actual Cd concentration 30 min after adding the contaminant reflected the nominal exposure concentration (1 µM = 112 µg/L), and the value decreased after 1 day. BaP concentration measured in the aquaria containing pristine MPs and MP-BaP was low. In the tank containing dissolved BaP, although markedly below the nominal exposure concentration (1 µM = 252 µg/L), BaP concentration was high 30 min after adding the contaminant and dropped notably after 1 day (Table 4).

### 3.3. Activity of Antioxidant and Peroxisomal Enzymes

**Figure 3:**
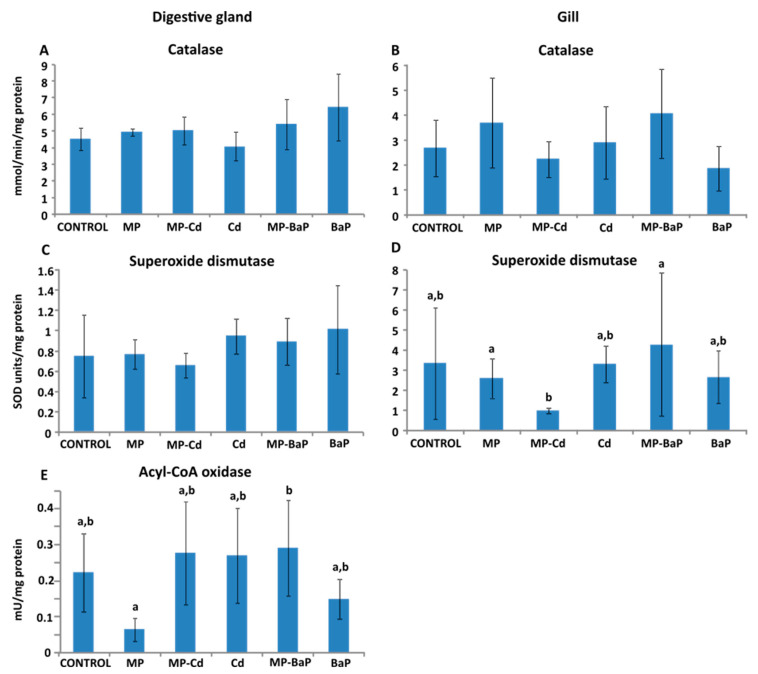
Activity of the antioxidant enzymes catalase in the digestive gland (**A**) and gills (**B**), superoxide dismutase in the digestive gland (**C**) and gills (**D**), and activity of acyl-CoA oxidase in the digestive gland (**E**) of mussels, presented as mean ± standard deviation (*n* = 6). Different letters indicate statistically significant differences (*p* < 0.05) according to the Tukey’s post hoc test after one-way ANOVA.

The highest CAT activity in the digestive gland was measured in organisms exposed to dissolved BaP and to MP-BaP (Figure 3A), with values of 6.431 ± 2.020 and 5.402 ± 1.497 mmol/min mg^−1^ protein, respectively. The lowest CAT activity was found in organisms exposed to dissolved Cd, as well as in the control organisms, with the mean values being 4.072 ± 0.873 and 4.523 ± 0.649 mmol/min mg^−1^ protein, respectively. Gill samples (Figure 3B) of organisms exposed to MP-BaP showed the highest CAT activity (4.053 ± 1.797 mmol/min mg^−1^ protein), whilst groups treated with dissolved BaP and MP-Cd expressed the lowest activity (1.868 ± 0.903 and 2.236 ± 0.720 mmol/min mg^−1^ protein, respectively). No statistically significant differences were found among the CAT activities measured in the digestive gland or gills of control and treated mussels.

The lowest SOD activity in the digestive gland (Figure 3C) was measured in organisms exposed to MP-Cd and in control organisms, with mean values of 0.659 ± 0.127 and 0.751 ± 0.409 units/min mg^−1^ protein, respectively. The highest activity was measured after BaP exposure, with 1.013 ± 0.437 units/min mg^−1^ protein, followed by those exposed to dissolved Cd, with 0.948 ± 0.171 units/min mg^−1^ protein. Assessing the gill samples (Figure 3D), the lowest SOD activity was also measured in organisms exposed to MP-Cd (0.973 ± 0.13 units mg^−1^ protein), followed by those exposed to pristine MPs, with 2.582 ± 0.967 units mg^−1^ protein. Here, the highest mean activity was also observed in mussels exposed to MP-BaP, with 4.276 ± 3.557 units mg^−1^ protein. The SOD activity measured in the gills was found to be significantly influenced by the treatment ($\chi^{2}$(5) = 15.656, *p* = 0.008). Post hoc testing determined that mussels exposed to MP-Cd presented significantly lower activity than mussels exposed to pristine MPs (*p* = 0.019) and mussels exposed to MP-BaP (*p* = 0.003).

The lowest mean AOX activity in the digestive gland samples (Figure 3E) was measured in mussels exposed to pristine MPs (0.149 ± 0.056 mU mg^−1^ protein), while the highest activity was observed in organisms exposed to the contaminated MPs. Significant differences were obtained (F (5,28) = 3.048 and *p* = 0.025), caused by the difference between the treatment groups exposed to pristine MPs and MP-BaP (Tukey HSD post hoc: *p* = 0.043).

### 3.4. Lysosomal Membrane Stability

**Figure 4:**
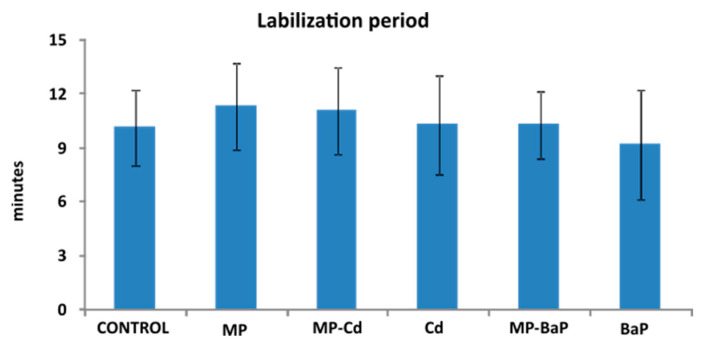
Labilization period (LP) of the digestive cell lysosomes. Mean ± standard deviation (*n* = 10). Statistically significant differences were not found according to the Kruskal–Wallis test (*p* < 0.05).

Overall, all experimental groups, including control mussels, showed low labilization period (LP) values (Figure 4). The mean LP value measured in BaP-exposed mussels (9.16 ± 3.06 min) was the lowest of all groups. The longest LP was found in organisms exposed to pristine MPs (11.25 ± 2.43 min). No statistically significant differences were found among experimental groups.

### 3.5. Tissue Metal Distribution and Accumulation after Autometallography

**Figure 5:**
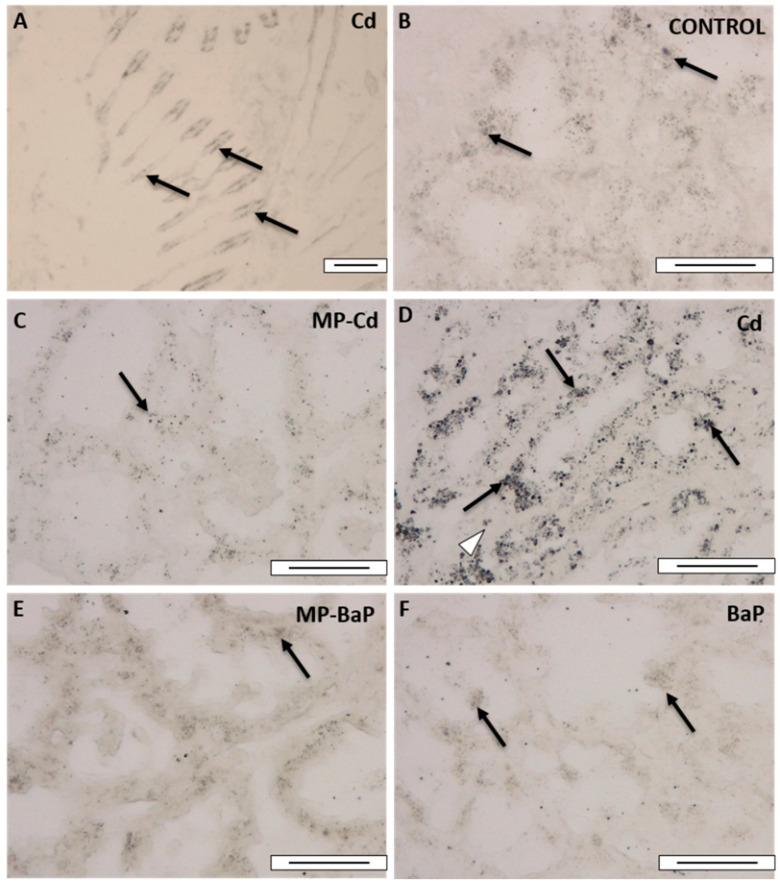
Micrographs of the gills (**A**) and digestive gland (**B**–**F**) of mussels after autometallographical staining. (**A**) Mussel exposed to 1 µM Cd for 3 days; (**B**) control mussel; (**C**) mussel exposed to 1000 particles/mL 4.5 µm MP-Cd for 3 days; (**D**) mussel exposed to 1 µM Cd for 3 days; (**E**) mussel exposed to 1000 particles/mL 4.5 µm MP-BaP for 3 days; (**F**) mussel exposed to 1 µM BaP for 3 days. Black silver deposits indicate the presence of metals in the gill cells (black arrows in **A**), in the digestive tissue (black arrows in **B**–**F**) and haemocytes (white triangle in **D**). Scale bars: 50 µm.

**Figure 6:**
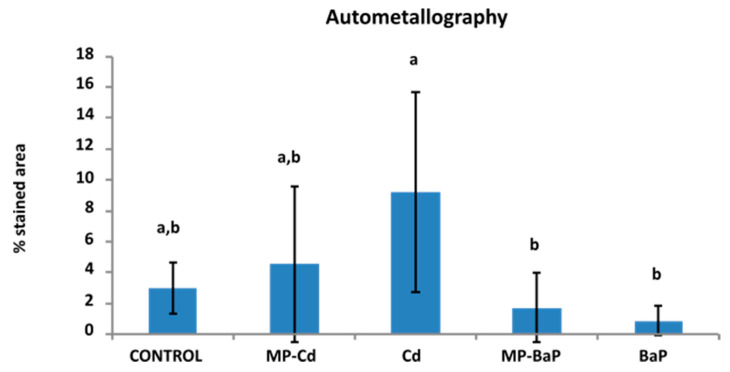
Results of the quantitative analysis of the autometallographical staining of the digestive gland. Mean ± standard deviation (*n* = 7–10). Different letters indicate statistically significant differences (*p* < 0.05), according to the Dunn’s post hoc test after performing a one-way Kruskal–Wallis test.

Metals revealed as BSDs after autometallographical staining (Figure 5) were mainly detected in the frontal zone of the gill filaments (Figure 5A) as well as in the digestive gland epithelium (Figure 5B–F). Occasionally, metals were also detected in the digestive gland haemocytes (Figure 5D). Since the main area for metal accumulation was the epithelium of the digestive tubules, the measurement of the percentage of tissue area that showed BSDs was focused in that area. As expected, results indicated that highest values were observed in mussels exposed to dissolved Cd (Figure 5D and Figure 6), while the lowest were observed in mussels exposed to dissolved BaP (Figure 6). The Kruskal–Wallis test showed a $\chi^{2}$(4) = 27.449 with *p* = 0.000, and the post hoc Dunn’s test showed significant differences between mussels exposed to dissolved Cd and those exposed to dissolved BaP and to MP-BaP (*p* = 0.000 and 0.016, respectively).

### 3.6. Quantitative Histological Analysis

**Figure 7:**
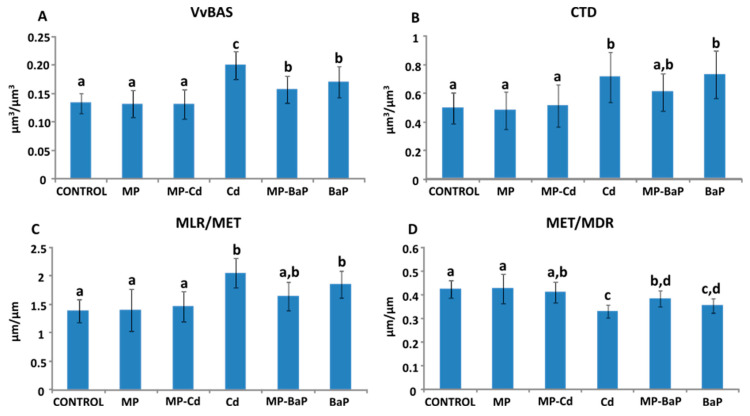
Results of the quantitative histological analysis of the structure of the digestive gland. (**A**) Volume density of basophilic cells; (**B**) connective-to-diverticula ratio; (**C**) mean luminal radius to mean epithelium thickness; (**D**) mean epithelium thickness to mean diverticular radius. Mean ± standard deviation (*n* = 10). Different letters indicate statistically significant differences (*p* < 0.05) according to the Tukey’s post hoc test after one-way ANOVA.

The volume density of basophilic cells (Figure 7A) had the highest values in mussels exposed to Cd (0.199 ± 0.024 µm^3^/µm^3^) and lowest in individuals exposed to MP-Cd (0.13 ± 0.026 µm^3^/µm^3^). One-way ANOVA indicated significant differences among experimental groups ($\chi^{2}$(5) = 13.422 with *p* = 0.000). The post hoc test showed that control mussels and mussels exposed to pristine MPs and to MP-Cd presented significantly lower values of VvBAS than the rest of exposed mussels. Mussels exposed to Cd showed significantly higher values than the other treatments. Similarly, tissue integrity (CTD) presented a similar trend to that shown by VvBAS (Figure 7B) with the lowest values observed in control mussels and those exposed to pristine MPs and MP-Cd, while mussels exposed to MP-BaP displayed intermediate values and mussels exposed to Cd and to BaP showed significantly higher values ($\chi^{2}$(5) = 5.955 with *p* = 0.000).The MLR/MET and MET/MDR ratios (Figure 7C,D) also presented a similar trend to that observed in VvBAS and CTD. Overall, control mussels and those exposed to pristine MPs and MP-Cd presented the lowest MLR/MET and highest MET/MDR values. The highest values for MLR/MET (2.04 ± 0.26 µm/µm) and lowest values in MET/MDR (0.33 ± 0.027 µm/µm) were measured in Cd exposed mussels. In both cases, significant differences were observed among experimental groups. In the case of MLR/MET ($\chi^{2}$(5) = 10.311 with *p* = 0.000), two statistical groups were distinguished with control mussels, mussels exposed to pristine MPs and to MP-Cd in one and mussels exposed to Cd and to BaP in other group, while mussels exposed to MP-BaP presented intermediate values. In the case of MET/MDR ($\chi^{2}$(5) = 9.563 with *p* = 0.000), control mussels, mussels exposed to pristine MP and to MP-Cd presented significantly higher values than mussels exposed to Cd and to BaP, and mussels treated with MP-BaP presented intermediate values (Figure 7D).

## 4. Discussion

A recent review regarding the applicability of mussels as global indicators for the coastal contamination by MPs concluded that the mussel provides great potential for global biomonitoring of both spatial and temporal international trends [26]. At least two ways of MP uptake, dependent on the particle size, have been described in mussels: via the gills involving microvilli and endocytosis and via the cilia that transferred MPs to the stomach and digestive gland [30]. A recent review further assessed the viability of the mussel digestive gland in terms of assessing anthropogenic pollutants, concluding that it is a reliable organ for cellular, molecular and biochemical assessment [61].

### 4.1. Quantitative Histological Analysis

Throughout the experiments, the selected MP particle concentrations were within reported environmentally relevant concentrations (e.g., 1770 particles/L found in the southern North Sea [53]), with the second experiment being designed based on the accumulation observed in the first experiment. This allows for the hypothesis that the results obtained in the present study may resemble natural occurrences. Most previous works are based on higher test concentrations to allow establishing effect concentrations [62,63,64], which however makes drawing conclusions for the aquatic ecosystem health more difficult. Studies have determined the uptake of MPs by marine mussels, as well as the organism’s ability to retain these particles for a length of time [31,33]. It was further shown that MPs are capable of being transferred through the food web [43,65,66], leading to increased concentrations in organisms higher in the trophic system, such as baleen whales [67] and the thorough assessment of the possible cellular and molecular effects of MP ingestion are thus of paramount importance.

The results of the first experiment determined that the digestive gland retained both 4.5 and 45 µm particles, even after a 3-day depuration period, whilst the 4.5 µm particles were observed in the gills more prominently than the 45 µm ones. In terms of digestive gland retention, these findings are in accordance with those of Gonçalves et al. [25], showing that 10 µm particles could be observed within the gut lumen but not the gills after 15 min of exposure. Long-term MP exposure (21 days) further showed that particles accumulated in the diverticula of the stomach and digestive gland, whilst no particles were found in other organs, even after a 7-day depuration period. The study further found that ingested MPs passed through the entire digestive tract and were expelled with the organism’s faeces. The present findings of 45 µm particles being more evident in the stomach lumen and connective tissue were also supported by previous studies [32].

These findings overall indicate that MP ingestion occurs in a concentration and depuration-time dependent manner, concurrent with previous work [31]. Here it was also determined that the gills have a higher affinity to small particles, also concurrent with previous findings [23,33]. The longer prevalence of the 4.5 µm particles in the gills indicates a longer overall exposure time, as the trapped particles may be ingested even after the direct exposure has ended. It should thus be considered that, at environmentally relevant concentrations, mussels are able to rapidly (1 day) ingest the particles and translocate them into various tissues before depurating them over time. Moreover, 4.5 µm MPs crossing the digestive gland epithelia have been observed in this study after 3 days of exposure as well as in previous studies [33].

### 4.2. Accumulation and Effects of Contaminated MPs

Research carried out in the North Pacific Gyre found that different samples of seawater contained between 0.4 and 9 ng/L of PAHs, whilst sampled MP fragments contained PAH concentrations of 6 to 249 ng/g of plastic [68], indicating an accumulation of PAHs on plastic particles. The contaminant measurements within the mussel tissue samples carried out in the present work further suggest that BaP was more easily adsorbed to the PS plastic particles than cadmium. Similarly, contaminated MPs have been reported as negligible vector for mercury bioaccumulation in clams [69]. The mussel tissue contained Cd and BaP, according to the respective exposure groups and the concentration of contaminants within the aquaria water, decreased with exposure time, allowing the assumption that the removed quantity was, at least partially, taken up by the mussels. This statement was further supported by previous research, indicating that MPs exposed to pyrene over 6 days showed a concentration and time dependent adsorption, as well as then significantly increasing the pyrene body burden in exposed mussels by more than 13-fold [32]. Similarly, González-Soto et al. [33] reported an exposure time- and MP size-dependent accumulation of BaP in mussels after exposure for 7 and 26 days to BaP contaminated PS MPs of 0.5 and 4.5 µm. Pittura et al. [44] further showed that BaP readily adsorbed to LDPE MPs and increased the measured BaP concentration in the digestive gland after a 7-day exposure.

Having shown that mussels successfully accumulated MPs, this study further investigated the variable effect that both pristine and contaminated MPs can have on the organism. First, several enzyme activities were assessed as a response to environmental stressors. Lowered antioxidant activities have been considered an indicator for overwhelmed antioxidant defences or an inability to remove reactive oxygen species [70]. A recently published review stated that MP ingestion frequently challenged the oxidative state of invertebrates and seemingly required an upregulation of the antioxidant system in response [71], further supporting the application of these markers in studies on the impact of MPs. However, the exposure to pristine MP particles has frequently failed to induce significant responses in antioxidant levels [32,44,72,73].

In the present study, catalase activity in the digestive gland and gills was not significantly impacted. A previous study found, however, that a 7-day exposure of mussels to 500 µg/L BaP decreased catalase activity, followed by an increase in activity after 21 days [74]. Furthermore, a study assessing the effects of MP pollution and ocean acidification on mussels determined that of the assessed antioxidant biomarkers only catalase activity was significantly increased with increased MP concentration [73]. Revel et al. [75] also showed that exposure to 10 µg/L MPs for 26 days significantly increases both catalase and SOD activity in the digestive gland of mussels. In the present work, SOD activity only varied significantly for the gill samples of organisms exposed to MP-Cd (lowered activity) in comparison to those exposed to both pristine and MP-BaP. This result suggested that a longer exposure time could be needed to affect the overall SOD activity in mussels.

In the present study, peroxisomal AOX activity decreased significantly in the digestive gland of organisms exposed to pristine MPs compared to those exposed to MP-BaP. Even though lipid metabolism, where peroxisomes play a key role, has been highlighted as a relevant target for MPs pollution [76], recent work has shown that no significant difference in AOX activity in the digestive gland was measured when exposed to either pristine or MP-BaP as well as dissolved BaP over 7 days [44]. These findings were further supported with a recent study conducted with oysters (*Crassostrea gigas*) [72]. Work performed by Orbea and Cajaraville [50] found that mussels inhabiting or transplanted to sites polluted by PAHs showed increased AOX activity. However, lab studies where mussels were exposed to BaP yielded controversial results. Orbea et al. [74] found a significant decrease of AOX activity in the digestive gland of mussels waterborne exposed for 1 day, while no changes were seen after 7 and 21 days of treatment. Cancio et al. [77] did not observe alteration of AOX activity after 1 day of BaP injection, but increased activity was registered after 7 days.

Lysosomal membrane stability (LMS) can be used not only as a diagnostic biomarker for lysosomal stress, but also for the prognosis of the animals’ health status [78]. The measured labilization periods (LPs) were overall lower than expected indicating, a possible disturbed health status of all mussels. The presence of relevant concentrations of some pollutants, such as Zn or PAHs (mainly naphthalene), at levels that have been described in moderately polluted areas [79] may be responsible of the relatively low stability of the lysosome membrane. Moreover, the data may also indicate that other stressing factors, such reproductive status, could be triggering the response, or that Plentzia is not as clean site as previously thought. Other pollutants in addition to PAHs and metals should be considered in future works. Similar levels of LP have been previously described in mussels from the same area exposed in the lab during similar exposure time (96 h [80]) The influence of feeding during the experimental period in lysosomal compartment that could lead into changes in both lysosomal size and lysosomal membrane stability [81] should also be considered. No differences in LP were found among exposure groups whereas longer exposure conditions to BaP contaminated MPs along with dissolved BaP led to significantly decreased membrane stability in a time dependent manner [44]. These differences could be due to differences in exposure periods or to different lysosome population measurement.

Regarding tissue metal distribution and accumulation after autometallography, it became evident that Cd accumulated mainly in the gills and the digestive gland. The percentage of BSDs in the cells followed the expected trend, where samples of organisms exposed to dissolved Cd showed a larger positively stained area followed by those exposed to MP-Cd, in agreement with results obtained by analytical chemistry. These results indicate that autometallography can be a suitable technique to detect the exposure to metal-contaminated MPs.

The present study demonstrated that the structure of the digestive gland was only impacted by the 3-day exposure to both dissolved BaP and MP-BaP, as well as Cd. The VvBAS significantly increased in mussels exposed to BaP and MP-BaP, whilst the two dissolved contaminants caused a significant increase of the CTD and MLR/MET ratios. Moreover, Cd exposure significantly reduced MET/MDR ratio, which also occurred, to a lesser degree, after exposure to dissolved BaP and to MP-BaP. The increase in VvBAS has previously been determined as an indicator of environmental stressors [82]. The effects exerted on cell type composition of the digestive gland, however, can be reversed, as shown by a study conducted after the Prestige oil spill in 2002. It was found that mussels affected by the contamination showed signs of recovery after two years [83]. Current results indicated that, although control mussels presented moderate levels of stress [60], in agreement with LP data, the presence of Cd, BaP and MP-BaP induced higher levels of stress. Moreover, the CTD values indicative of the structural integrity of the digestive gland tissue, with a high ratio indicating reduced digestive tissue [59], suggest that both Cd and BaP and the MP-BaP induced a reduction of digestion capability, which may disrupt the normal functioning of the organism in the long-run. This was further supported by the MLR/MET and MET/MDR values, where an increase of the first parameter and decrease on the second is indicative of epithelial thinning due to stressors [59,60], a response that has previously been observed in mussels exposed to the water accommodated fraction of oils [84], metals [85] or MPs [33]. The fact that control mussels present some altered biomarker responses (relatively low LP and high VvBAS) could be indicative that selected season (spring; developing gametes) and site (Plentzia) could be optimized for future research, as commented before. Longer exposure-times could confirm whether present alterations are transitory of are confirmed and increased after exposure. Conversely, although generally for dissolved contaminants biochemical changes precede histological ones [29], higher alterations were observed at tissue level compared with biochemical measurements. Similarly, the Manila clam (*Ruditapes philippinarum*) was found to ingest MPs and whilst none of the assessed biochemical biomarkers showed significant responses after 7-day exposure, the histological assessment of individuals exposed to MPs alone or co-exposed with Hg indicated deterioration of the gill epithelial tissue along with haemocyte infiltration [69].

## 5. Conclusions

Results of the present work demonstrated that marine mussels ingest MPs of various sizes (4.5 and 45 µm) and that these particles can further be accumulated in the digestive gland in a concentration and depuration time dependent manner. Furthermore, it was found that BaP body burdens increased notably in mussels exposed to MP-BaP, making it evident that plastic debris with adsorbed contaminants are posing a threat to the marine wildlife. This research was carried out over a 3-day period, which would only indicate initial impacts of exposure. Many factors may influence the impact that plastics and contaminants may have on organisms, of which not all are known or fully understood yet. Activity of the antioxidant and peroxisomal enzymes did not show a clear response to MP exposure, but autometallography appeared as a suitable technique to detect the exposure to metal-contaminated MPs. Quantitative histological analysis allowed for the determination of stress caused by the exposure to BaP and Cd and to MP-BaP by determination of changes in the basophilic cells volume density and the connective-to-diverticula ratio, as well as two ratios indicating digestive tubule structure. The results of this research make it evident that more work is needed in this field, as there are still knowledge gaps in the understanding of contaminants and their association with plastic debris, as well as their impact on marine organisms at long-term.

## Figures and Tables

**Table 1:** Prevalence of mussels presenting MPs and abundance of particles found in the different structures of the digestive gland and in the gills. Data are expressed as mean ± standard deviation.

Group			Digestive Gland						Gills			
			*n*	F	Stomach Lumen	Duct Lumen	Tubule Lumen	Connective Tissue	*n*	F	within Filaments	outside Filaments
**45 µm particles**	**C1**	**E**	10	50	0.3 ± 0.67	n.o.	n.o.	0.1 ± 0.3	10	20	n.o.	0.3 ± 0.67
		**D1**	10	10	0.3 ± 0.95	0.1 ± 0.32	n.o.	n.o.	9	n.o.	n.o.	n.o.
		**D2**	10	n.o.	n.o.	n.o.	n.o.	n.o.	10	n.o.	n.o.	n.o.
		**D3**	10	n.o.	n.o.	n.o.	n.o.	n.o.	10	n.o.	n.o.	n.o.
	**C2**	**E**	8	100	8.1 ± 7.41	3.5 ± 8.75	0.25 ± 0.71	1.37 ± 2.39	10	30	n.o.	0.8 ± 1.62
		**D1**	10	n.o.	n.o.	n.o.	n.o.	n.o.	10	20	n.o.	0.2 ± 0.42
		**D2**	10	20	1.1 ± 2.33	0.1 ± 0.32	n.o.	n.o.	10	n.o.	n.o.	n.o.
		**D3**	10	20	0.2 ± 0.63	n.o.	n.o.	n.o.	10	10	n.o.	0.1 ± 0.32
	**C3**	**E**	10	100	83.6 ± 112.32	13 ± 27.02	1.1 ± 1.91	11.7 ± 31.60	10	40	n.o.	1.1 ± 2.18
		**D1**	10	90	16.7 ± 21.71	2.4 ± 3.47	n.o.	0.3 ± 0.67	10	20	0.1 ± 0.32	0.4 ± 0.97
		**D2**	10	60	9.1 ± 21.75	3.2 ± 9.77	0.7 ± 2.21	2.3 ± 5.66	10	20	n.o.	0.3 ± 0.67
		**D3**	10	40	5.1 ± 11.73	0.1 ± 0.32	n.o.	n.o.	10	10	n.o.	0.1 ± 0.32
**4.5 µm particles**	**C1**	**E**	10	50	0.4 ± 0.63	0.3 ± 0.67	n.o.	n.o.	10	50	0.4 ± 0.7	1 ± 1.05
		**D1**	10	30	0.3 ± 0.48	0.1 ± 0.32	n.o.	n.o.	10	40	0.1 ± 0.32	0.9 ± 1.29
		**D2**	10	20	0.1 ± 0.32	0.1 ± 0.32	n.o.	n.o.	10	20	0.1 ± 0.32	0.5 ± 0.97
		**D3**	9	n.o.	n.o.	n.o.	n.o.	n.o.	10	20	0.1 ± 0.32	0.1 ± 0.32
	**C2**	**E**	9	70	1.7 ± 1.41	0.9 ± 0.93	0.7 ± 0.71	n.o.	10	80	0.5 ± 0.71	2.7 ± 1.95
		**D1**	10	50	0.6 ± 0.84	0.3 ± 0.48	0.2 ± 0.42	0.1 ± 0.32	10	70	0.4 ± 0.70	1.9 ± 1.59
		**D2**	10	50	0.2 ± 0.42	0.2 ± 0.42	0.2 ± 0.42	n.o.	10	40	0.3 ± 0.67	0.6 ± 0.97
		**D3**	10	20	0.1 ± 0.32	n.o.	n.o.	n.o.	10	40	0.2 ± 0.42	0.4 ±0.70
	**C3**	**E**	10	90	1 ± 0.82	0.9 ± 0.74	0.9 ± 0.74	0.3 ± 0.48	10	90	0.8 ± 0.79	2.8 ± 1.75
		**D1**	10	80	0.2 ± 0.42	0.7 ± 0.82	0.5 ± 0.53	0.1 ± 0.32	10	70	0.8 ± 0.79	1.4 ± 1.43
		**D2**	10	50	0.4 ± 0.52	0.3 ± 0.48	0.2 ± 0.42	n.o.	10	60	0.3 ± 0.48	1.1 ± 1.20
		**D3**	10	30	0.1 ± 0.32	0.1 ± 0.32	0.1 ± 0.32	n.o.	9	40	0.6 ± 1.01	0.6 ± 0.88

E: exposure group; D: depuration groups; F: prevalence (%); n: number of examined individuals; n.o: no particles observed.

**Table 2:** Results of the chemical analyses of mussels sampled in Plentzia (Bay of Biscay) in March 2016. Data are expressed as mean ± standard deviation.

**Metal**	**µg/g dw**
Fe	141 ± 22.06
Zn	446.6 ± 93.75
Cr	1.72 ± 0.16
Ni	1.52 ± 0.34
Cu	4.62 ± 0.128
Cd	0.65 ± 0.07
Pb	1.95 ± 0.21
**PAH**	**ng/g dw**
Naphthalene	1555.8 ± 253.56
Acenaphthylene	5.6 ± 2.07
Acenaphthalene	bdl
Fluorene	5 ± 2
Phenanthrene	25.6 ± 1.52
Anthracene	111.6 ± 168.45
Fluoranthene	54 ± 5.48
Pyrene	63.6 ± 9.96
Benzo(a)anthracene	31 ± 3.16
Chrysene	57.2 ± 4.44
Benzo(b)fluoranthene	33 ± 4.64
Benzo(k)fluoranthene	26.2 ± 4.33
Benzo(a)pyrene	81.8 ± 35.05
Indeno pyrene	<6 ± 2 *
Dibenzo(a,h)anthracene	<3.67 ± 1.5 *
Benzo(ghi)perylene	14.4 ± 4.16
**TOTAL PAHs**	**<2071.5**

bdl: below detection limit; dw: dry weight; * values for some of the replicates were bdl and those samples were not used to calculate mean values.

**Table 3:** Results of the chemical analyses of Cd and BaP concentrations in mussels exposed to pristine or contaminated MPs, or to dissolved contaminants. Data are expressed as mean ± standard deviation.

Group	Cd (µg/g dw)	BaP (ng/g dw)
MP	0.59 ± 0.09	18.5 ± 16.25
MP-Cd	0.60 ± 0.16	nm
Cd	33 ± 7.85	nm
MP-BaP	nm	3050 ± 777.82
BaP	nm	192,450 ± 11,101.58

dw: dry weight; nm: not measured for this sample set.

**Table 4:** Results of the chemical analyses of Cd and BaP concentrations in water samples of the second experiment. Samples were collected 30 min and 1 day after each dosing. Data are expressed as mean ± standard deviation.

Group	Cd (µg/L)		BaP (µg/L)	
	30 min	1 day	30 min	1 day
MP	bdl	bdl	0.03 ± 0.03	0.12 ± 0.10
MP-Cd	bdl	bdl	nm	nm
Cd	112.33 ± 2.08	74.37 ± 7.99	nm	nm
MP-BaP	nm	nm	0.30 ± 0.29	0.29 ± 0.13
BaP	nm	nm	40.60 ± 31.71	1.72 ± 1.45

bdl: below detection limit; nm: not measured for this sample set.

## Data Availability

Data are contained within the article.
